# Bulk Modification of Wood Using a Combination of Hydrophobic Silica Sol–Gel and Nanoclay to Enhance Dimensional Stability and Durability

**DOI:** 10.3390/ma19214503

**Published:** 2026-10-26

**Authors:** Miklós Bak, Dávid Takács

**Affiliations:** Faculty of Wood Engineering and Creative Industries, University of Sopron, 9400 Sopron, Hungary; takacs.david@uni-sopron.hu

**Keywords:** wood modification, vinyltriethoxysilane, Cloisite 30B, nanoclay, dimensional stability, fungal resistance, bulk impregnation

## Abstract

Wood modification aimed at reducing moisture sensitivity and biological degradation should improve dimensional stability and durability without unacceptable deterioration of mechanical performance. This study investigated bulk impregnation of European beech (*Fagus sylvatica* L.) and Scots pine (*Pinus sylvestris* L.) using vinyltriethoxysilane (VTES), Cloisite 30B nanoclay (C30B), and NaOH, and the combination of VTES and C30B with emphasis on the different NaOH concentrations (VTES + CN-1, VTES + CN-2, and VTES + CN-3). Treatment uptake and retention, volumetric bulking coefficient, dimensional stability, mechanical properties, decay resistance, color, and FTIR spectral changes were evaluated. The combined CN treatments provided the greatest dimensional stabilization. VTES + CN-2 produced the highest volumetric anti-swelling efficiency, reaching 60.51% in beech and 60.05% in Scots pine. Longitudinal compressive strength was not significantly affected, although hardness decreased after some treatments. The CN formulations also reduced fungal mass loss in both species by up to 40.9% compared to the control. Overall, the results show that combined VTES-C30B-NaOH modification can improve dimensional stability and biological resistance, but its effectiveness depends strongly on formulation and wood species.

## 1. Introduction

Wood is a renewable material with a favorable strength to weight ratio and offers a wide range of structural and non-structural applications. However, its hygroscopic nature limits its performance under varying environmental conditions. Water sorption and desorption by cell wall polymers cause dimensional changes, while prolonged exposure to high humidity increases the risk of fungal decay. Repeated swelling and shrinkage may also lead to deformation, surface cracking, and a reduced service life of wood products [1,2]. Consequently, wood modification processes generally aim to reduce moisture sensitivity and improve biological durability without causing unacceptable deterioration in mechanical or aesthetic properties [2,3,4].

Organosilicon compounds have been investigated as wood-modifying agents because they can be introduced into wood by impregnation and subsequently undergo hydrolysis and condensation. Depending on their molecular structure and the treatment conditions, alkoxysilanes may be deposited in the cell lumina, penetrate the cell wall, or form condensed structures on the internal surfaces of wood [3,4]. Previous studies have reported reduced liquid water uptake, improved water repellency, and increased dimensional stability following treatment with various silanes. The effectiveness of these treatments was influenced by the functional groups, concentration, and hydrolysis state of the silane, the wood species, and the post-treatment conditions applied [3,4,5]. Amino-functional silanes have also been associated with increased resistance to wood-decaying fungi, although these effects cannot be generalized to all organosilicon compounds [6].

The mechanism responsible for improvements in moisture-related properties is not necessarily limited to chemical reactions between silanes and wood polymers. Deposited or condensed treatment agents may reduce the accessible pore volume, increase the cell-wall volume, or partially restrict cell-wall swelling [3,7]. In wood treated with methyltrimethoxysilane, the reduction in equilibrium moisture content, after accounting for the retained silane content, was attributed primarily to an increase in cell-wall volume rather than solely to a reduction in the number of available hydroxyl groups [7]. Similarly, the effectiveness of organosilane treatments has been shown to depend on the moisture content of the wood and the conditions applied during condensation [8]. These findings suggest that weight gain and spectroscopic changes alone are insufficient to confirm a specific modification mechanism. When evaluating wood–water interactions, the location and physical state of the retained material should also be taken into account [7,8].

The incorporation of nanoscale inorganic materials represents an additional potential approach to improving wood properties. Nanoclay impregnation has been shown to reduce water uptake and swelling and to modify the mechanical and thermal behavior of solid wood, although the magnitude of these effects depends on the concentration and distribution of the nanoclay [9]. Montmorillonite-based nanoclays are particularly relevant because their layered structure and surface hydroxyl groups allow further modification with organofunctional silanes. Vinyltriethoxysilane has been used for the silanization of montmorillonite, and the resulting modification was influenced by the reactivity of the clay surface, the properties of the silane, the dispersion medium, and the reaction conditions [10]. However, observations from clay–polymer systems cannot be directly applied to solid wood, where anatomical structure, permeability, and cell-wall accessibility influence the uptake and distribution of treatment agents [9,10].

Studies on vinyl-functionalized inorganic particles suggest that the performance of silane-containing systems depends not only on their chemical composition but also on the distribution and morphology of the deposited phase. Xue et al. produced superhydrophobic films from silica nanoparticles modified with vinyltriethoxysilane and found that the resulting wettability was governed primarily by the disordered micro- and nanoscale surface structure and the associated air fraction, rather than by surface chemistry alone [11]. Jia et al. subsequently applied a one-step treatment containing vinyltriethoxysilane, silica nanoparticles, and sodium hydroxide to wood and reported that the amount of alkali influenced particle deposition, surface roughness, wettability, and the mechanical durability of the resulting layer [12]. These findings suggest that alkaline conditions may influence silane hydrolysis and condensation, as well as the distribution and retention of silane-containing inorganic phases.

Silica- and silane-based treatments have already demonstrated that nanoscale inorganic structures can improve the dimensional stability and moisture-related properties of both hardwoods and softwoods [13]. At the same time, these effects may be accompanied by color changes, uneven retention, or species-dependent performance [3,13]. The combination of vinyltriethoxysilane and Cloisite 30B nanoclay may influence clay dispersion, treatment retention, and the condensed structures formed within the wood [10,12,13]. Alkaline conditions may further affect the formation of the treatment system. The resulting wood properties therefore cannot be predicted solely from the presence or amount of individual components [9,11,12,13]. A systematic comparison of mixtures containing different proportions of vinyltriethoxysilane, Cloisite 30B, and NaOH is therefore needed.

Despite the available information on silane-modified wood and nanoclay impregnation, only limited data are available on the combined use of vinyltriethoxysilane and Cloisite 30B in solid wood [3,4,5,6,7,8,9,10,11,12,13]. Cloisite 30B was involved in the investigation as a nanoparticle because of its availability and relatively low price. On the other hand, functionalization (hydrophobization) of silica nanoparticles by VTES is already known through its available hydroxyl groups. Cloisite 30B has similarly available hydroxyl groups which make this nanoparticle a good source of functionalization by VTES. In particular, the effects of formulation composition on treatment uptake, dimensional stabilization, moisture uptake, mechanical properties, color, and biological resistance have not yet been evaluated together in anatomically different hardwood and softwood species. Such a comparison is necessary because treatment uptake and the resulting changes in properties may differ considerably between species, even when identical formulations and impregnation conditions are used.

Therefore, the aim of the present study was to evaluate the effects of impregnation formulations containing vinyltriethoxysilane, Cloisite 30B, and NaOH on European beech (*Fagus sylvatica* L.) and Scots pine (*Pinus sylvestris* L.). The expectation was that NaOH concentration has a strong influence on the effect of the bulk treatment of wood. Thus, leaving the ratio of the other components constant, NaOH was varied in the treatment system using three different concentrations. Treatments containing the individual components and their combinations were compared with untreated wood. The investigation included treatment uptake and retention, wood–water relations, dimensional stability, mechanical and optical properties, resistance to fungal decay, and treatment-related changes in the FTIR spectra. The hypothesis was that the combined formulations would reduce moisture sensitivity and improve dimensional and biological performance compared with untreated wood and wood treated with comparison treatments, while the magnitude of these effects and any associated property trade-offs would depend on both formulation composition and wood species. Such improvements could enhance the suitability of wood for construction purposes, particularly in outdoor applications where dimensional stability and durability are important.

## 2. Materials and Methods

### 2.1. Materials and Specimen Preparation

European beech (*Fagus sylvatica* L.) and Scots pine sapwood (*Pinus sylvestris* L.) were used in this study. The specimens were selected randomly. The wood originated from growing sites in Hungary.

The specimens were prepared so that their principal edges were parallel to the anatomical directions of the wood. For most measurements, specimens with dimensions of 20 mm × 15 mm × 20 mm (R × T × L) were used, including the mass gain, dimensional stability, and mechanical tests. Specimens measuring 15 mm × 20 mm × 50 mm were used for the fungal decay test and 5 mm × 10 mm × 30 mm for FTIR measurements.

Absolute ethanol (VWR Ltd., Debrecen, Hungary), Cloisite 30B nanoclay (C30B, Rockwood Clay Additives GmbH, Moosburg an der Isar, Germany), sodium hydroxide solution (96%; VWR Ltd., Debrecen, Hungary), and vinyltriethoxysilane (VTES, Sigma-Aldrich Co., LLC., Budapest, Hungary) were used for the wood modification procedure. The concentration of the NaOH solution prepared with distilled water was adjusted so that the NaOH concentration in the VTES + NaOH, VTES + CN-1, VTES + CN-2, and VTES + CN-3 treatment solutions was 0.1, 0.1, 0.4, and 0.5 mol/L, respectively.

### 2.2. Wood Modification Treatment

The quantities used to prepare the wood modification solutions are shown in Table 1. The components were mixed on a magnetic stirrer at 500 rpm for 10 min. The prepared solution was then homogenized using a homogenizer (WiseTis HG-15A, Witeg Labortechnik GmbH, Germany) at maximum speed for 30 min.

The prepared solution was then poured over the specimens, and the specimens were impregnated with the solution. The vacuum stage of the impregnation was carried out at 100 mbar and 20 °C for 30 min. The specimens were then left in the solution at atmospheric pressure and 20 °C for 2 h. After removal from the solution, excess liquid was removed from the specimen surfaces using paper towels, and the specimen mass was recorded. The specimens were then placed in a drying oven at 60 °C for 24 h. Subsequently, they were dried to an oven-dry state at 103 ± 2 °C.

### 2.3. Test Methods

Depending on the test method, the specimens were either conditioned at 20 °C and 65% relative humidity or dried to an oven-dry state at 103 ± 2 °C. The specimen dimensions were measured using a Mitutoyo ID-C150XB digital indicator (0.001 mm, Mitutoyo Corporation, Tokyo, Japan). Measurements were taken at the same point in each case, at the center of the specimen surface. The specimen mass was measured using a Precisa XT 1220M-FR balance (0.001 g, Precisa Gravimetrics AG, Dietikon, Switzerland).

Solution uptake (SU) and weight percentage gain (WPG) were determined during the tests. SU and WPG were calculated using Equations (1) and (2), respectively. Ten independent specimens were tested for each treatment group.

SU % = (m_w_ − m_0_)///m_0_ × 100, (1)

where m_w_ is the wet mass after impregnation (g), and m_0_ is the oven-dry mass before impregnation (g).

WPG % = (m_I0_ − m_0_)///m_0_ × 100, (2)

where m_I0_ is the oven-dry mass after impregnation (g), and m_0_ is the oven-dry mass before impregnation (g).

The volumetric bulking coefficient (VBC) of the treatments was characterized by the final change in specimen volume. Measurements were performed on oven-dry specimens. VBC was calculated using Equation (3). Ten independent specimens were tested for each treatment group.

VBC % = (V_I0_ − V_0_)///V_0_ × 100, (3)

where V_I0_ is the oven-dry volume after impregnation (mm^3^), and V_0_ is the oven-dry volume before impregnation (mm^3^).

The equilibrium moisture content (EMC), volumetric swelling (S_V_), and anti-swelling efficiency (ASE) of the specimens were determined. The specimen dimensions were measured in the oven-dry state and after conditioning at 20 °C and 65% relative humidity. Linear swelling was calculated separately in the radial, tangential, and longitudinal directions as the percentage difference between the conditioned and oven-dry dimensions relative to the oven-dry dimension. EMC was calculated using Equation (4), S_V_ using Equation (5), and ASE using Equation (6). Ten independent specimens were tested for each treatment group.

EMC_20/65_% = (m_I,20/65_ − m_I0_)///m_0_ × 100, (4)

where m_I,20/65_ is the mass of the impregnated specimen after conditioning at 20 °C and 65% relative humidity (g), and m_I0_ is the oven-dry mass after impregnation (g).

S_V_ % = (V_T,20/65_ − V_T0_)///V_T0_ × 100, (5)

where V_T,20/65_ is the volume of the treated specimen after conditioning at 20 °C and 65% relative humidity (mm^3^), and V_T0_ is the oven-dry volume of the treated specimen (mm^3^).

ASE % = (S_control_ − S_treated_)///S_control_ × 100, (6)

where S_control_ is the volumetric swelling of the untreated control specimens at 20 °C and 65% relative humidity, and S_treated_ is the volumetric swelling of the treated specimens under the same conditions.

Before the mechanical tests, the specimens were conditioned. The compressive strength test was performed in accordance with ISO 13061-17:2017 [14] using a universal testing machine (Instron 5985, Norwood, MA, USA). The specimens were loaded parallel to the grain. According to the standard, compressive strength was determined as the ratio of the maximum load to the initial cross-sectional area under load. The obtained values were corrected to 12% moisture content using Equation (7). Ten independent specimens were tested for each treatment group.

σ_12%_ = σ_comp_ × (1 + α × (MC–12)), (7)

where σ_comp_ is the compressive strength of the specimen conditioned at 20 °C and 65% relative humidity, MC is the moisture content of the specimen, and α is the correction factor (0.04).

Hardness was determined using the Brinell–Mörath method at a maximum load of 500 N. Measurements were performed in the radial, tangential, and end-grain surface. The measurements were taken at the center of the specimen faces, with one measurement in each anatomical direction. Ten independent specimens were tested for each treatment group. The obtained values were corrected to 12% moisture content using Equation (8).

H_B-M 12%_ = H_B-M_ × (1 + α × (MC–12)), (8)

where H_B-M_ is the hardness of the specimen conditioned at 20 °C and 65% relative humidity in the respective anatomical direction, MC is the moisture content of the specimen, and α is the correction factor (0.04 for the end-grain surface and 0.025 for the radial and tangential surfaces).

Resistance to fungal decay was evaluated according to CEN/TS 15083-1:2005 [15] based on percentage mass loss (ML). The white-rot fungus *Trametes versicolor* was used for European beech, while the brown-rot fungus *Coniophora puteana* was used for Scots pine. The exposure period was 16 weeks. Five specimens were tested for each treatment group. After exposure, surface mycelium was removed, and the specimens were dried at 103 ± 2 °C until constant mass was reached. One untreated specimen for each modified specimen served as control. A set of each series of sterile specimens were stored on agar medium without fungi culture to evaluate treatment loss during incubation. Mass loss was calculated using Equation (9).

ML % = (m_before_ − m_after_)///m_0f_ × 100, (9)

where m_before_ is the oven-dry mass before fungal exposure (g), m_after_ is the oven-dry mass after fungal exposure (g) and m_0f_ is the oven-dry mass before any treatment.

SEM-EDX mapping was performed at a depth of 10 mm from the surface to avoid the effect of agglomeration of treatment material on the surface. The surfaces of the 15 mm × 20 mm × 15 mm specimens were final-cut with a razor blade prior to imaging. SEM-EDX elemental mapping was performed at 5 regions of each sample. No sputter coating was applied to the specimen surfaces. SEM–EDX elemental mapping was performed using a Hitachi S-3400N instrument (Hitachi Ltd., Tokyo, Japan) at a chamber pressure of 70 Pa and an accelerating voltage of 10 kV.

Color was measured using a Konica Minolta CM-2600d spectrophotometer (Konica Minolta Inc., Tokyo, Japan) with d/8° measurement geometry, D65 illuminant, and an 8 mm measurement aperture. Measurements were performed at one location on each specimen, using 10 specimens per treatment, both before and after treatment. The CIELAB coordinates (L*, a*, and b*) were recorded. Color changes relative to the initial state were expressed as ΔL*, Δa*, Δb*, total color difference ΔE*, and change in chroma ΔC*.

Fourier-transform infrared spectra were recorded using a JASCO FT/IR-6300 spectrometer (JASCO Corporation, Tokyo, Japan) operated in DRIFT mode. The spectra were recorded over the range of 4000–400 cm^−1^ at a resolution of 4 cm^−1^, with 50 scans averaged for each spectrum. The spectra were processed using the Kubelka–Munk transformation. The resulting spectra were normalized at 1382 cm^−1^ for European beech and at 1378 cm^−1^ for Scots pine. For each treatment, spectra from three independent specimens were averaged, and difference spectra were calculated by subtracting the spectra recorded before treatment from those recorded after treatment. Spectral changes were evaluated primarily in the ranges of 3800–2600 cm^−1^ and 1900–800 cm^−1^.

Statistical significance tests were performed using Statistica 10.0 software (StatSoft), including analysis of variance (ANOVA) and Fisher’s LSD test (*p* < 0.05), to determine the effect of treatment on the investigated material properties.

## 3. Results and Discussion

### 3.1. Treatment Uptake and Permanent Bulking

**Figure 1:**
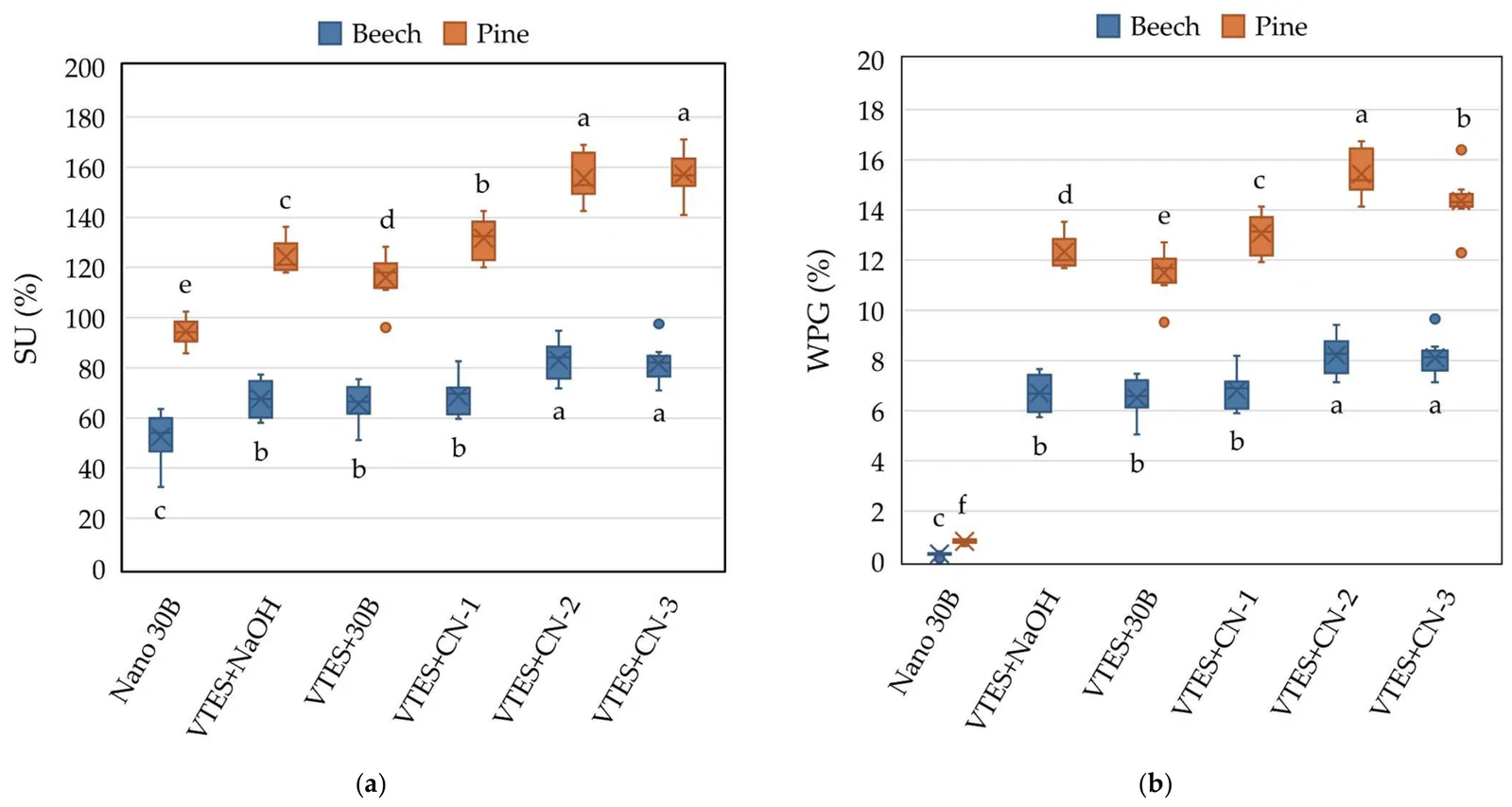
Effects of the different treatments on European beech and Scots pine, shown as (**a**) solution uptake (SU) and (**b**) weight percentage gain (WPG). Box-and-whisker plots show the distribution of the measured values; crosses indicate mean values and points outside the whiskers indicate outliers. Different lowercase letters indicate significant differences among treatments within the same wood species according to Fisher’s LSD test (*p* < 0.05). Letter groupings are not comparable between wood species.

To evaluate the effectiveness of the treatments, SU and WPG were examined in European beech and Scots pine specimens (Figure 1). The results showed that all treatment solutions significantly affected both SU and WPG. The two parameters followed similar trends regardless of wood species. However, for Scots pine, both values were consistently nearly twice those measured for European beech, which may be explained by differences in anatomical structure. An opposite trend was observed compared with our previous study on treatment with functionalized silica nanoparticles [16], with the present results indicating a higher impregnation level in Scots pine. In addition, WPG closely followed the changes in SU. On average, WPG accounted for approximately 9.92% of SU. The only exceptions were the Nano 30B treatments (European beech 0.58 ± 0.19%, Scots pine 0.85 ± 0.12%).

For European beech, the VTES + NaOH and VTES + 30B treatments did not differ significantly from the VTES + CN-1 treatment. This suggests that the alkaline environment and the addition of nanoclay alone did not affect treatment efficiency. In contrast, a higher SU value under alkaline conditions was observed for Scots pine.

For the VTES + CN treatments, changes in the NaOH concentration were associated with increases in SU and WPG, as the NaOH concentration increased. The cell-wall swelling effects of NaOH and water–ethanol mixtures are well known from the literature [17,18,19]. This effect may have resulted in greater treatment uptake and facilitated better incorporation of the components. For European beech, there was no significant difference between the VTES + CN-2 and VTES + CN-3 treatments. For Scots pine, the VTES + CN-3 treatment showed a smaller increase in WPG than the VTES + CN-2 treatment. These two observations may indicate that the NaOH concentration in the VTES + CN-2 treatment reached an optimum regarding WPG.

The exceptionally low WPG observed for the Nano 30B treatments was nevertheless consistent with the expected result considering the proportion of C30B in the treatment solution.

Higher values alone do not show where the treatment agent was retained. It may have entered the cell wall, but it may also have been deposited in the cell lumen or on the surface.

**Figure 2:**
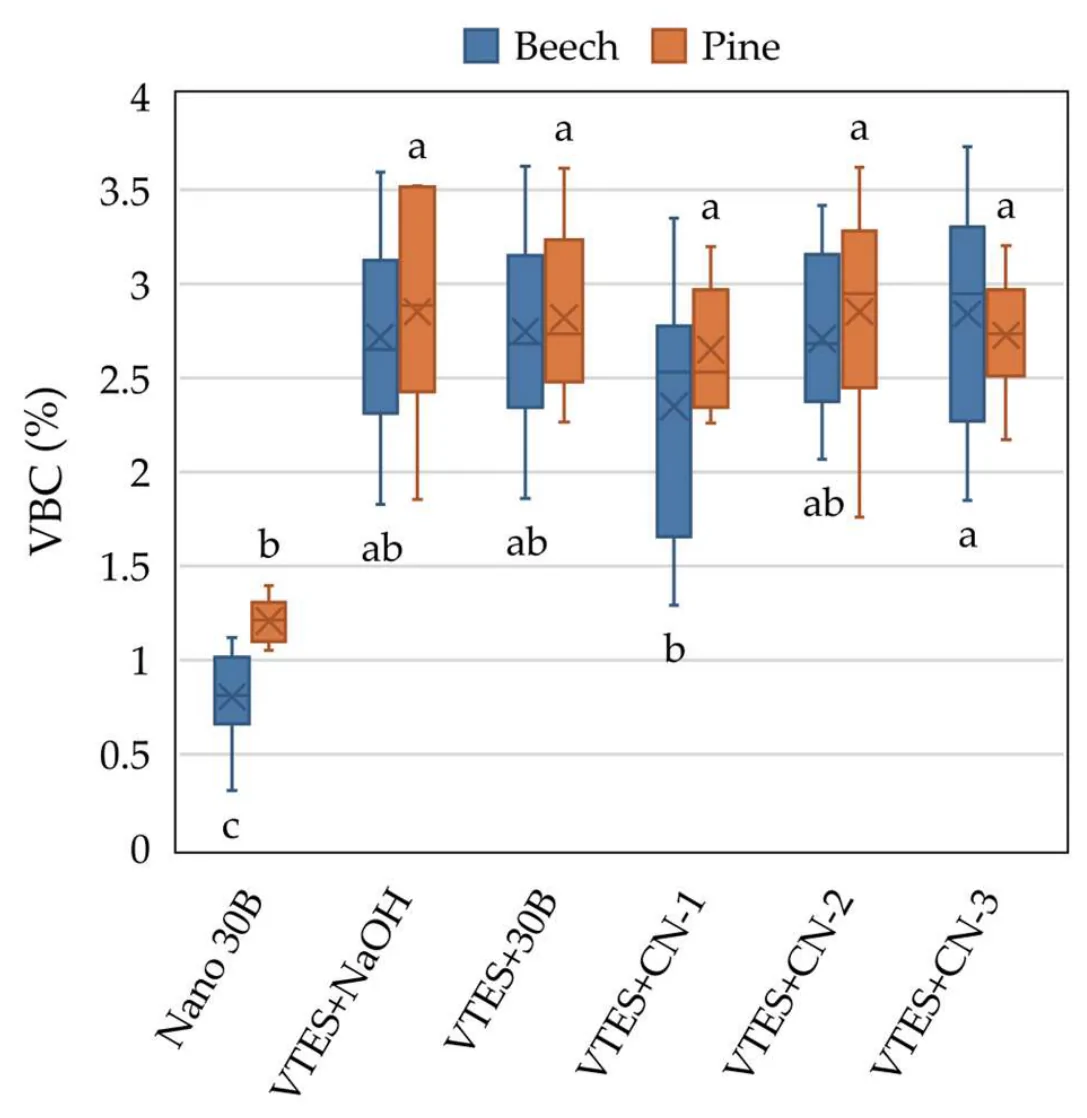
Volumetric bulking coefficient of European beech and Scots pine after the different treatments. Different lowercase letters indicate significant differences among treatments within the same wood species according to Fisher’s LSD test (*p* < 0.05). Letter groupings are not comparable between wood species.

The changes in permanent VBC induced by the treatments are shown in Figure 2. VBC increased significantly compared with the initial state for both wood species and all treatments. The Nano 30B treatment resulted in a relatively small permanent increase in cell-wall volume, with mean values of 0.80 ± 0.25% for European beech and 1.21 ± 0.12% for Scots pine. In contrast, the VTES-containing treatments resulted in substantially higher VBC values, ranging from 2.35 to 2.84% in European beech and from 2.65 to 2.85% in Scots pine. For European beech, the highest mean VBC was obtained with the VTES + CN-3 treatment, whereas the highest values in Scots pine were observed for VTES + NaOH and VTES + CN-2. It is important to note that, among the VTES-containing treatments in European beech, a statistically significant difference was observed only between VTES + CN-1 and VTES + CN-3.

Despite the previously observed low WPG, a small increase in VBC also occurred in Nano 30B-treated European beech. This is consistent with the possibility that part of the retained treatment agent reached the accessible pore system of the cell wall [3,20]. Overall, European beech and Scots pine responded similarly to the treatments in terms of VBC.

For the VTES-containing treatments, reducing the VTES concentration within the CN series did not result in a proportional decrease in VBC. This may indicate a non-linear relationship between the amount of retained treatment agent and volumetric bulking coefficient. Part of the additional material retention may instead result from deposition in the cell lumina or other macroscopic voids [21].

### 3.2. Dimensional Stability

**Figure 3:**
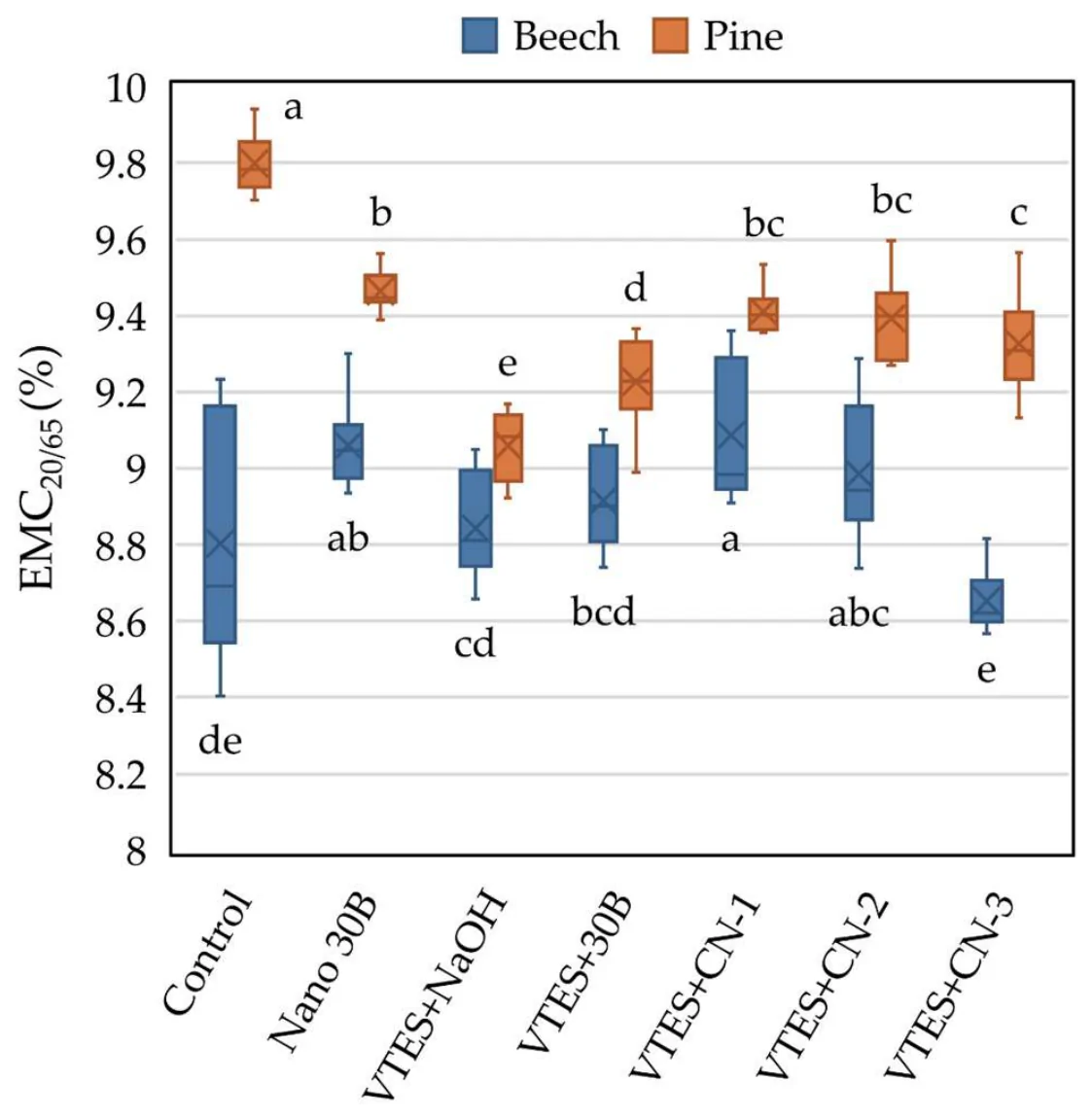
Equilibrium moisture content (EMC_20/65_) of beech and Scots pine at 20 °C and 65% relative humidity after the different treatments. Different lowercase letters indicate significant differences among treatments within the same wood species according to Fisher’s LSD test (*p* < 0.05). Letter groupings are not comparable between wood species.

To evaluate the hygroscopic behavior of the modified wood, equilibrium moisture content (EMC) was determined at 20 °C and 65% relative humidity (Figure 3). Treatment significantly affected EMC in both wood species (*p* < 0.001), although the direction and magnitude of the changes differed considerably between European beech and Scots pine. In European beech, EMC varied within a relatively narrow range of 8.65–9.09%. Compared with the untreated control (8.80 ± 0.32%), the Nano 30B, VTES + CN-1, and VTES + CN-2 treatments resulted in significantly higher EMC values, whereas VTES + NaOH and VTES + 30B did not differ significantly from the control. The lowest mean EMC was obtained with the VTES + CN-3 treatment (8.65 ± 0.08%). However, this value did not differ significantly from the untreated control, although it was significantly lower than those of all other treated groups.

In contrast, all treatments significantly reduced the EMC of Scots pine compared with the untreated control (9.80 ± 0.08%), with mean values ranging from 9.06 to 9.46%. This corresponded to a reduction of approximately 3.4–7.5% relative to the control. The lowest EMC was observed after the VTES + NaOH treatment (9.06 ± 0.09%), which was significantly lower than that of all other treatments. No significant differences were detected among the three CN formulations.

The different responses of the two wood species indicate that the effect of the treatments on EMC was strongly species-dependent. Previous bulk treatments with hydrophobic silica nanoparticles and in situ formed silica aerogel generally reduced EMC in both European beech and Scots pine, although the magnitude of the moisture-related response and water-vapor transport also depended on wood species and treatment system [13,22]. Thus, the absence of an overall reduction in EMC in European beech in the present study suggests that the hygroscopic effect cannot be attributed solely to the presence or amount of silane- or silica-containing phases. More generally, the moisture sorption capacity of wood depends on the accessibility of sorption sites and the structural state of the cell wall [1]. According to previous studies, alkoxysilane treatments may alter these properties through cell-wall penetration and bulking, while the magnitude of this effect depends on the molecular size and condensation state of the silane species [3,20]. Accordingly, the differences among the present treatments may be associated with species-dependent penetration and distribution of the modifying agents.

**Figure 4:**
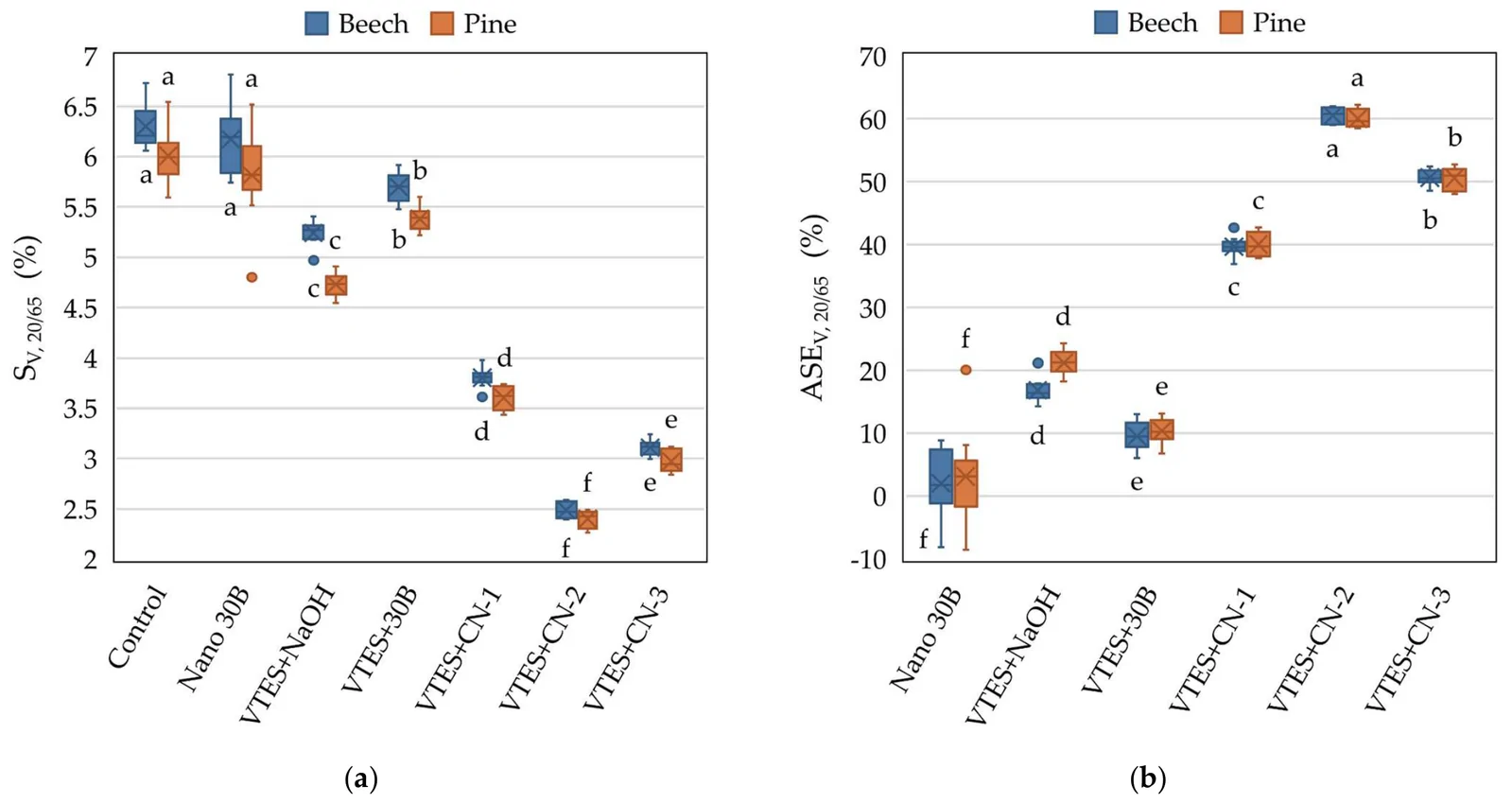
Effects of the different modification treatments on (**a**) volumetric swelling (S_V,20/65_), and (**b**) volumetric anti-swelling efficiency (ASE_V,20/65_) of modified beech and Scots pine at 20 °C and 65% relative humidity.

The volumetric swelling of the untreated control specimens (Figure 4a) was 6.30 ± 0.23% for European beech and 6.01 ± 0.27% for Scots pine. The Nano 30B treatment reduced volumetric swelling only to a limited extent and did not differ significantly from the untreated control in either wood species. In contrast, the VTES + NaOH and VTES + 30B treatments resulted in significantly lower volumetric swelling, although their dimensional stabilization effect was considerably weaker than that achieved by the combined application of VTES, C30B, and NaOH. The VTES + NaOH treatment resulted in volumetric ASE values of 16.82% and 21.23%, while the VTES + 30B treatment resulted in values of 9.54% and 10.39% for European beech and Scots pine, respectively (Figure 4b). Treatments containing all three components produced substantially greater dimensional stability. The VTES + CN-1 treatment also resulted in an ASE_V_ of approximately 40% in both wood species. The greatest dimensional stabilization effect was obtained with the VTES + CN-2 treatment, for which volumetric swelling decreased to 2.49 ± 0.08% in European beech and 2.40 ± 0.09% in Scots pine; these values corresponded to ASE_V_ values of 60.51% and 60.05%, respectively. However, a further increase in NaOH concentration did not enhance the effect, as ASE_V_ decreased to approximately 51% with the VTES + CN-3 treatment.

The substantial improvement in dimensional stability cannot be explained solely by changes in VBC or EMC, because treatments resulting in comparable VBC values showed markedly different ASE_V_ responses. This suggests that, in addition to volumetric bulking coefficient and equilibrium moisture content, the distribution and retention of the treatment agents may also have contributed to restricting swelling [3,20].

The limited effect of Nano 30B alone, together with the substantially higher ASE_V_ values obtained with the three-component formulations, suggests that the combined presence of VTES, C30B, and an alkaline environment was important for dimensional stabilization. The highest ASE_V_ was achieved with the VTES + CN-2 combination, indicating an optimum of NaOH concentration within the investigated formulation series.

### 3.3. SEM–EDX Analysis

**Figure 5:**
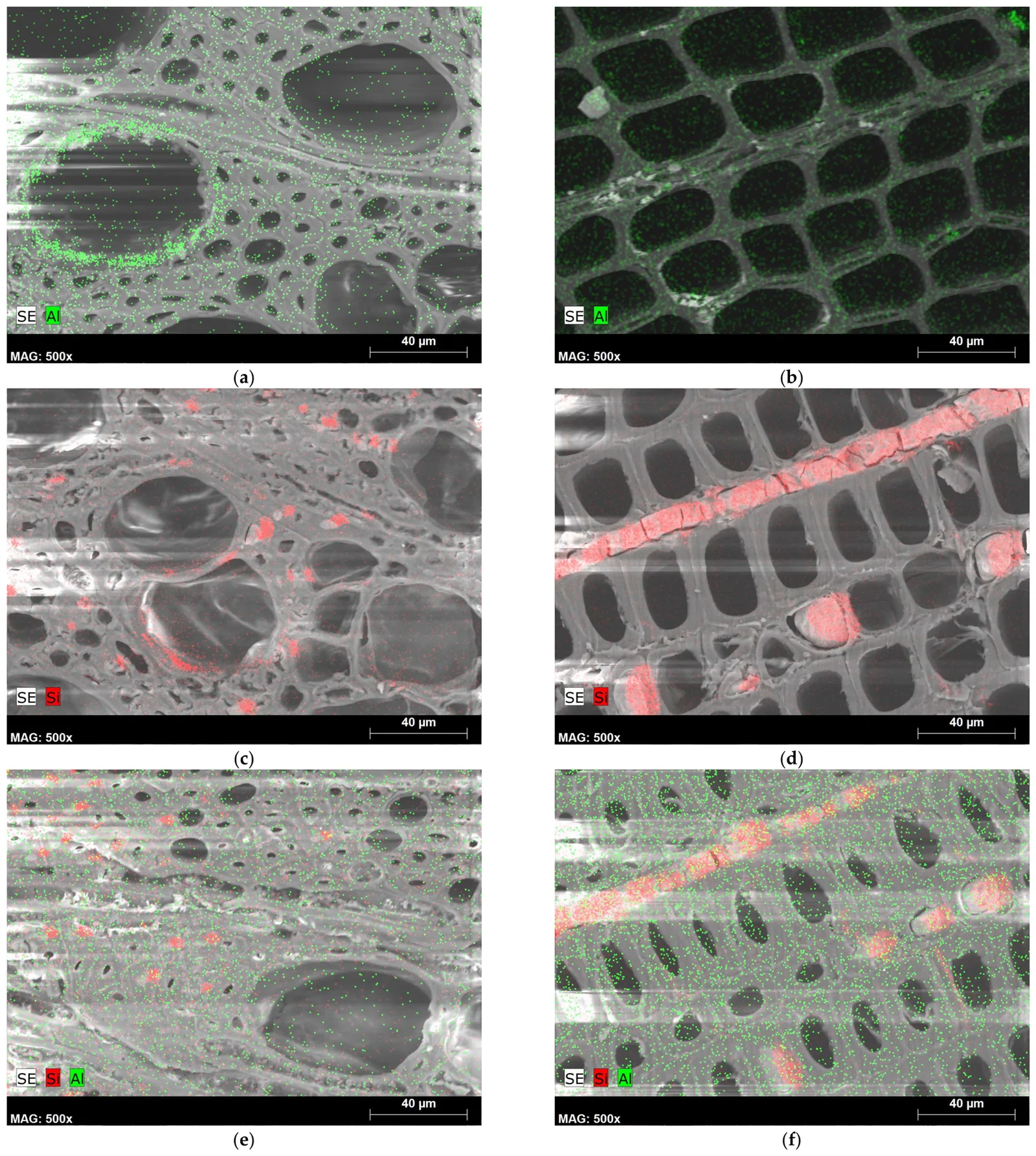

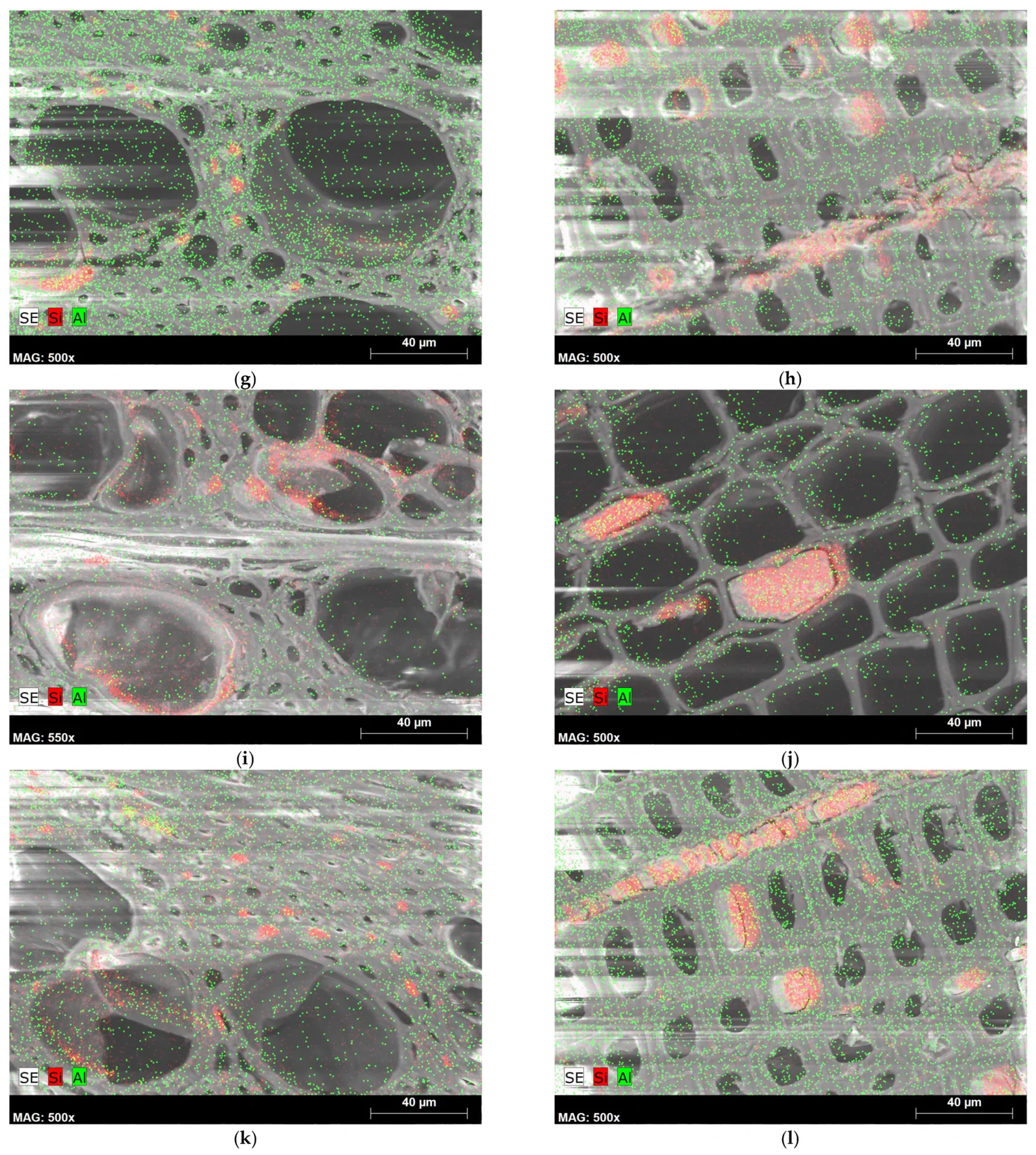
SEM–EDX silicon (Si) elemental maps of treated European beech and Scots pine. European beech is shown in the left column and Scots pine in the right column: (**a**,**b**) Nano 30B, (**c**,**d**) VTES + NaOH, (**e**,**f**) VTES + 30B, (**g**,**h**) VTES + CN-1, (**i**,**j**) VTES + CN-2, and (**k**,**l**) VTES + CN-3. The Si signal is shown in red over the secondary-electron images. Magnification: 500×; scale bar: 40 μm.

SEM–EDX mapping revealed Si signals in the analyzed areas of all treated European beech and Scots pine specimens, indicating the presence of Si-containing treatment agents. In both species, Si was widely distributed and was detected both in regions corresponding to the cell walls and within the cell lumina (Figure 5).

In European beech, the cell-wall-associated Si signal was predominantly localized toward the outer cell-wall regions, particularly in areas corresponding to the primary cell wall and middle lamella. A similar pattern was observed in Scots pine; however, Si signals were also detected in regions of the secondary cell wall. This difference may be associated with species-specific anatomical differences that influence the penetration pathways and spatial distribution of the treatment agents [19]. Nevertheless, the spatial resolution of SEM–EDX does not allow the detected Si to be assigned unambiguously to individual cell-wall layers.

**Figure 6:**
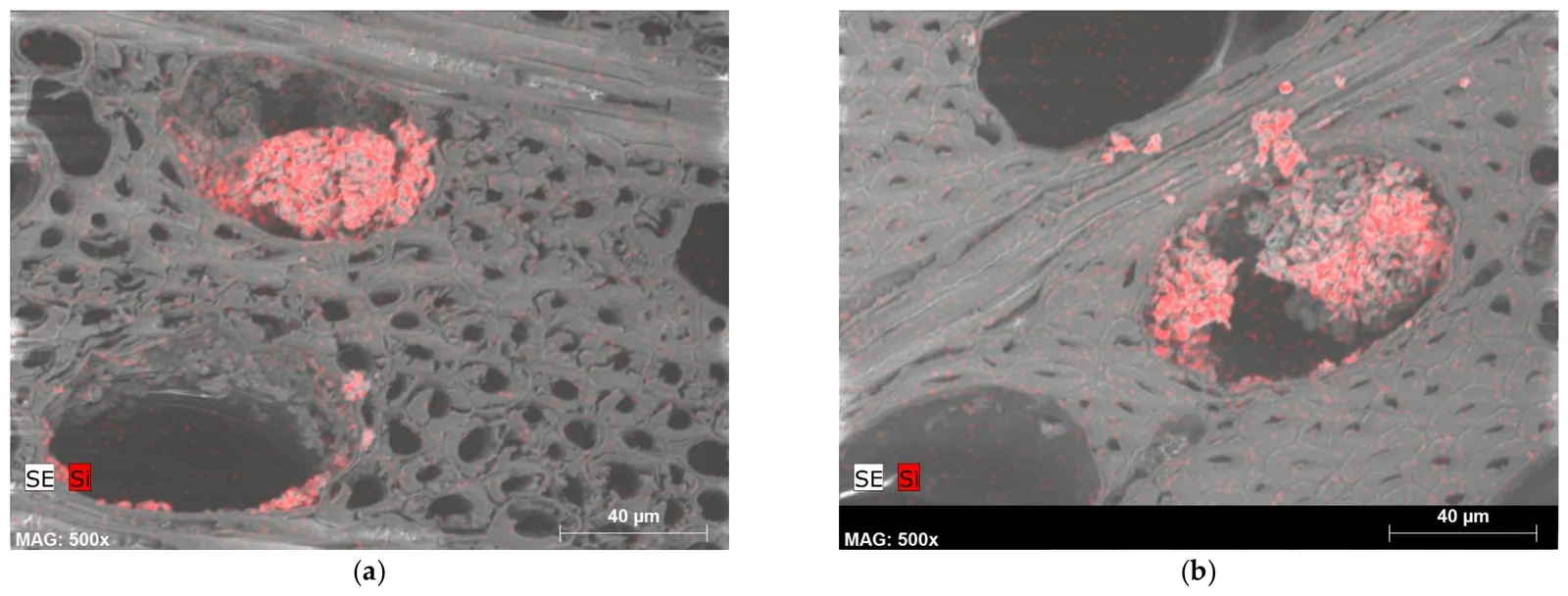
Representative localized Si-rich deposits in vessel lumina of European beech after (**a**) Nano 30B and (**b**) VTES + CN-3 treatments. The Si signal is shown in red over the secondary-electron images. Magnification: 500×; scale bar: 40 μm.

Extensive filling of the cell lumina was generally not observed, as it was expected based on the relatively low WPG (0.58–15.44%). In Scots pine, pronounced Si-rich material within the tracheid lumina was found mainly after the VTES + NaOH treatment (Figure 5d). No clearly separated, isolated Si-rich agglomerates were observed in the examined Scots pine sections. In contrast, localized Si-rich accumulations were observed in several European beech specimens, ranging from relatively small deposits (Figure 5k) to larger accumulations occupying a substantial part of individual vessel lumina (Figure 6). These localized accumulations occurred together with the more widely distributed Si.

### 3.4. Mechanical Properties

**Figure 7:**
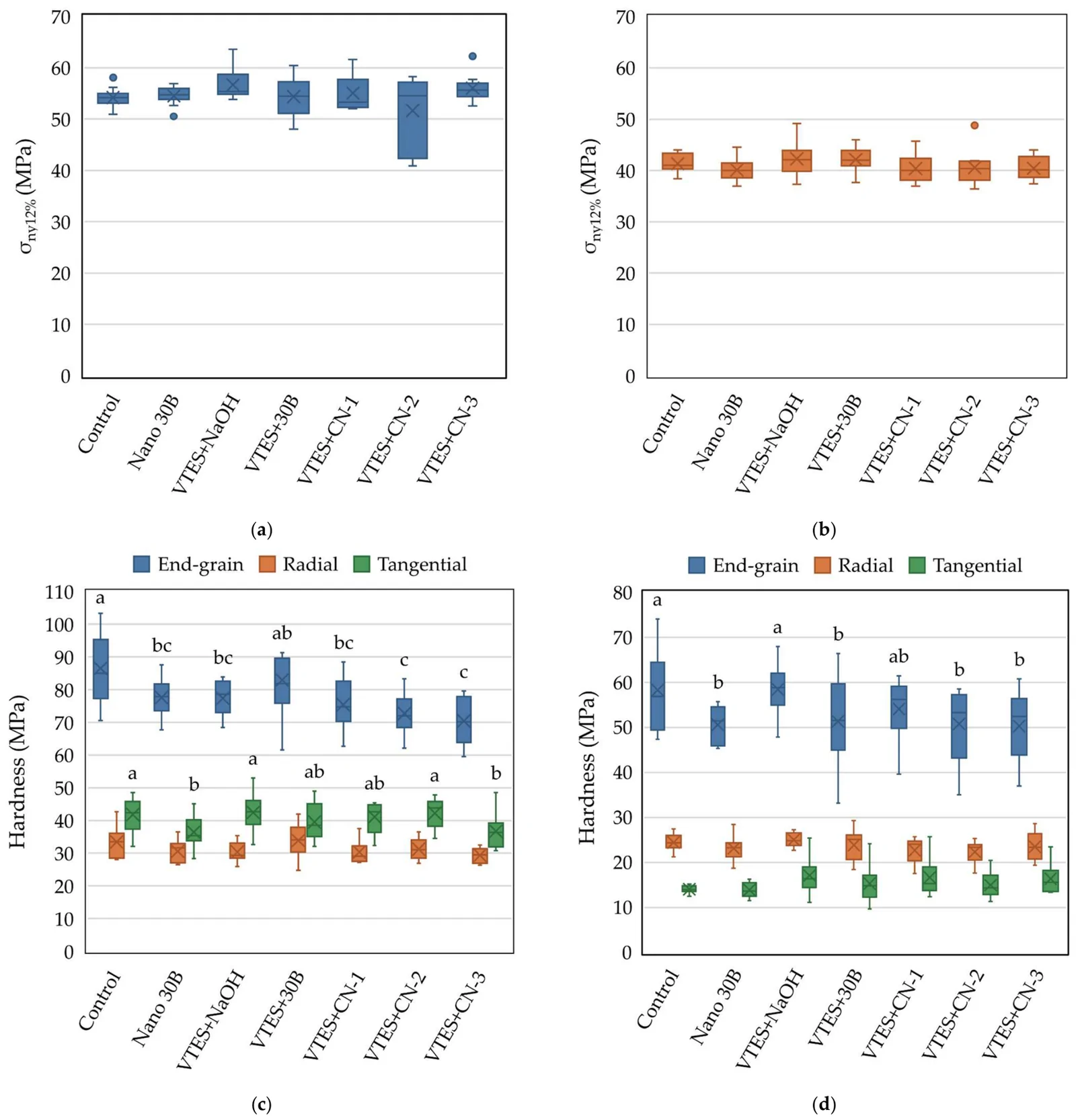
Mechanical properties of European beech and Scots pine after the different modification treatments: longitudinal compressive strength corrected to 12% moisture content for (**a**) beech and (**b**) Scots pine, and Brinell–Mörath hardness corrected to 12% moisture content for (**c**) European beech and (**d**) Scots pine, measured on end-grain, radial, and tangential surfaces. Different lowercase letters indicate significant differences among treatments within the same wood species and measurement direction according to Fisher’s LSD test (*p* < 0.05).

The treatments did not significantly affect compressive strength in either wood species (Figure 7a,b). Mean values ranged from 51.66 to 56.67 MPa for European beech and from 39.80 to 42.26 MPa for Scots pine. The absence of a significant reduction in compressive strength can be considered a favorable result, because organosilicon treatments, particularly under alkaline conditions, may reduce compressive strength parallel to the grain depending on the treatment conditions [23].

In both wood species, the treatment effect was mainly observed in end-grain hardness (Figure 7c,d). In European beech, most treatments significantly reduced end-grain hardness compared with the control, whereas the VTES + 30B treatment did not differ significantly from it. In Scots pine, Nano 30B, VTES + 30B, VTES + CN-2, and VTES + CN-3 resulted in significantly lower end-grain hardness. VTES + NaOH did not differ from the control, while VTES + CN-1 formed an intermediate group. Radial hardness was not significantly affected by the treatments in either wood species. In European beech, tangential hardness was significantly lower after the Nano 30B and VTES + CN-3 treatments than in the control and in the VTES + NaOH and VTES + CN-2 groups. In Scots pine, no significant treatment effect was observed on this surface.

The reduction in hardness was mainly confined to the end-grain surface, while radial hardness remained statistically unchanged in both wood species. Tangential hardness in Scots pine also remained unchanged in statistical terms. The effect of the treatments on the tested mechanical properties was selective, which is consistent with previously reported treatment-system-dependent mechanical responses of organosilicon- and nanoclay-based treatments [21,24]. Compressive strength parallel to the grain was maintained, while local surface hardness decreased slightly for certain treatments and anatomical surfaces.

### 3.5. Resistance to Fungal Decay

**Figure 8:**
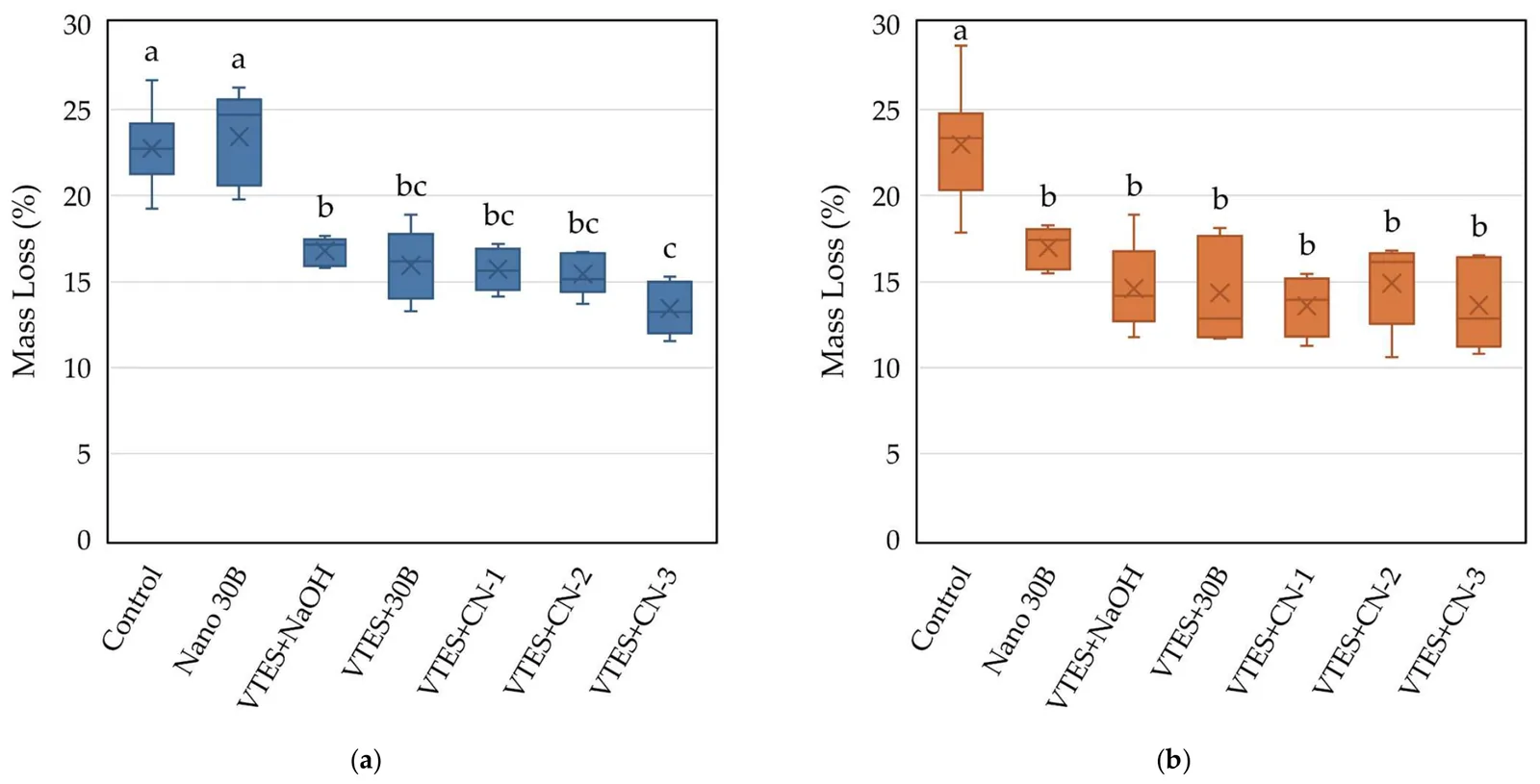
Mass loss of (**a**) European beech specimens exposed to *Trametes versicolor* and (**b**) Scots pine specimens exposed to *Coniophora puteana* for 16 weeks. Different lowercase letters indicate significant differences among treatments within each wood–fungus system according to Fisher’s LSD test (*p* < 0.05).

During fungal exposure, European beech exposed to *Trametes versicolor* showed a clear treatment-dependent response (Figure 8a). Specimens stored on sterile agar medium without fungi culture did not show treatment loss during the 16 weeks of incubation. The untreated control showed a mass loss of 22.75 ± 2.56%, while the Nano 30B treatment resulted in a similar value (23.42 ± 2.72%) and did not differ significantly from the control. In contrast, all VTES-containing treatments significantly reduced mass loss, with mean values ranging from 13.44 to 16.79%. The lowest mass loss was measured for the VTES + CN-3 treatment (13.44 ± 1.55%), corresponding to a 40.9% reduction relative to the untreated control. VTES + CN-3 showed significantly lower mass loss than VTES + NaOH, whereas it did not differ significantly from VTES + 30B, VTES + CN-1, or VTES + CN-2. Thus, in the European beech–*T. versicolor* relation, the reduction in mass loss was associated with the VTES-containing treatments, while C30B alone did not result in a detectable reduction.

A different response was observed for Scots pine exposed to *Coniophora puteana* (Figure 8b). The untreated control showed a mass loss of 22.99 ± 3.22%, whereas all treated groups showed significantly lower values ranging from 13.58 to 16.99%. No significant differences were found among the treated groups. Descriptively, the Nano 30B treatment resulted in the highest mean mass loss (16.99 ± 1.22%), whereas the lowest values were measured for VTES + CN-1 (13.58 ± 1.75%) and VTES + CN-3 (13.62 ± 2.65%). However, these numerical differences were not statistically significant. Specimens stored on sterile agar medium without fungi culture did not show treatment loss during the 16 weeks of incubation.

Based on the decay tests using *T. versicolor* and *C. puteana* the classification according to EN 350:2016 [25] for the treatments Nano 30B, VTES + NaOH, VTES + 30B, VTES + CN-1 and VTES + CN2 is durability class 4 (DC4—slightly durable), while it is DC3 (moderately durable) for VTES + CN-3. This classification also shows that there is a significant improvement in durability, but the treatment itself does not provide full protection for wood. Our results are consistent with mass-loss values reported for wood modified with certain organosilicon compounds. The VTES-containing treatments of European beech exposed to *T. versicolor* resulted in mass losses of 13.44–16.79%. Pries et al. reported a mass loss of 14.2% for European beech treated with a combination of acetylation and an acetoxy-functional siloxane [26]. In the Scots pine–*C. puteana* relation, the VTES-containing treatments resulted in mass losses of 13.58–14.90%, while Reinprecht and Grznárik [27] reported 9.2% for Scots pine treated with VTMS. The absolute mass loss was lower in the latter study, but the mass loss of untreated control was also lower (15.6%) than in the present study (22.99%). Therefore, the relative reduction compared with the control was of a similar magnitude, approximately 41% for VTMS in the study by Reinprecht and Grznárik and 35% for the VTES-containing treatments in the present study. For complex evaluation on the effect of decay resistance, tests with more fungal species in combination with leaching test are necessary.

### 3.6. Colour Changes

**Figure 9:**
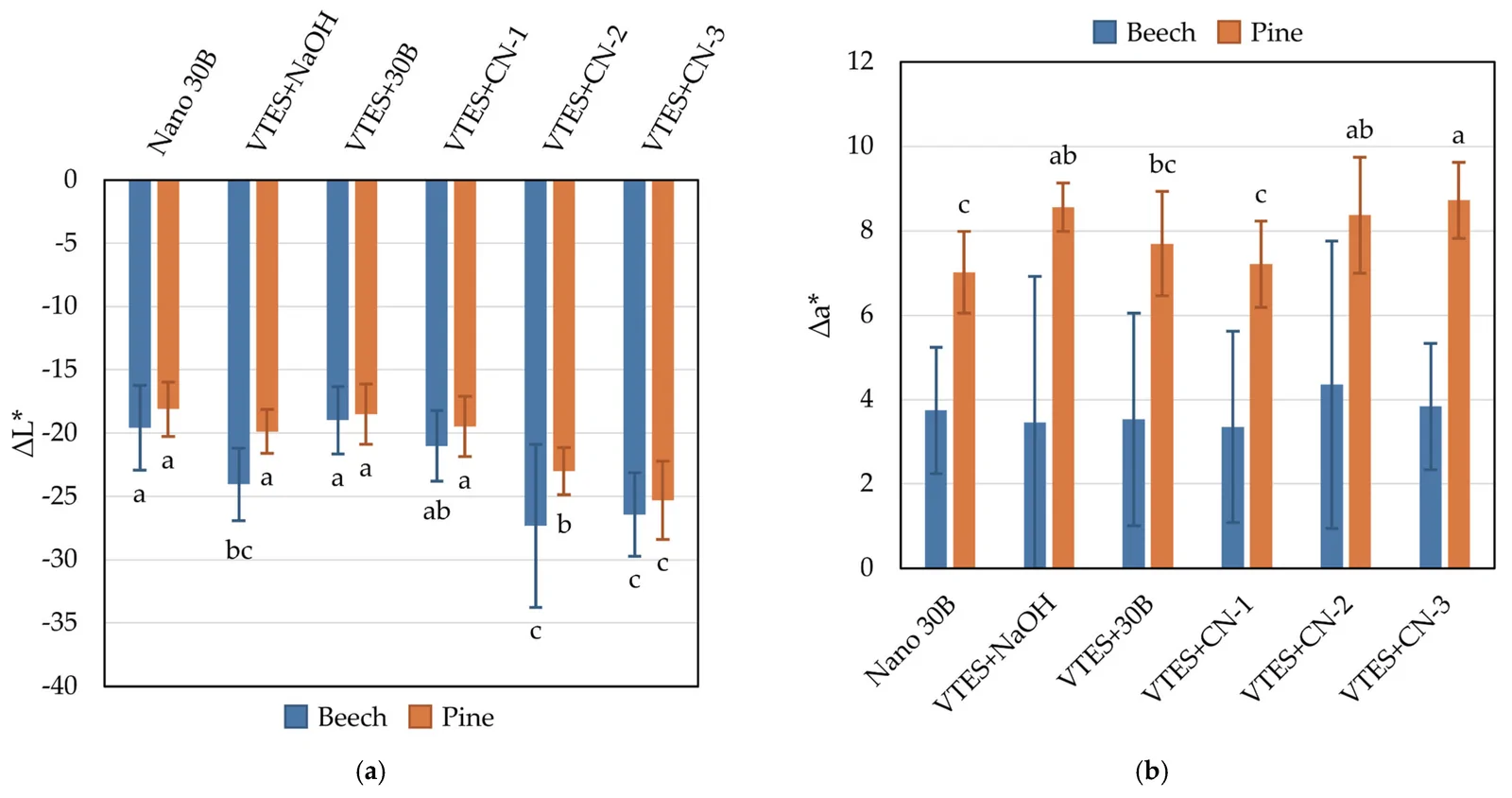

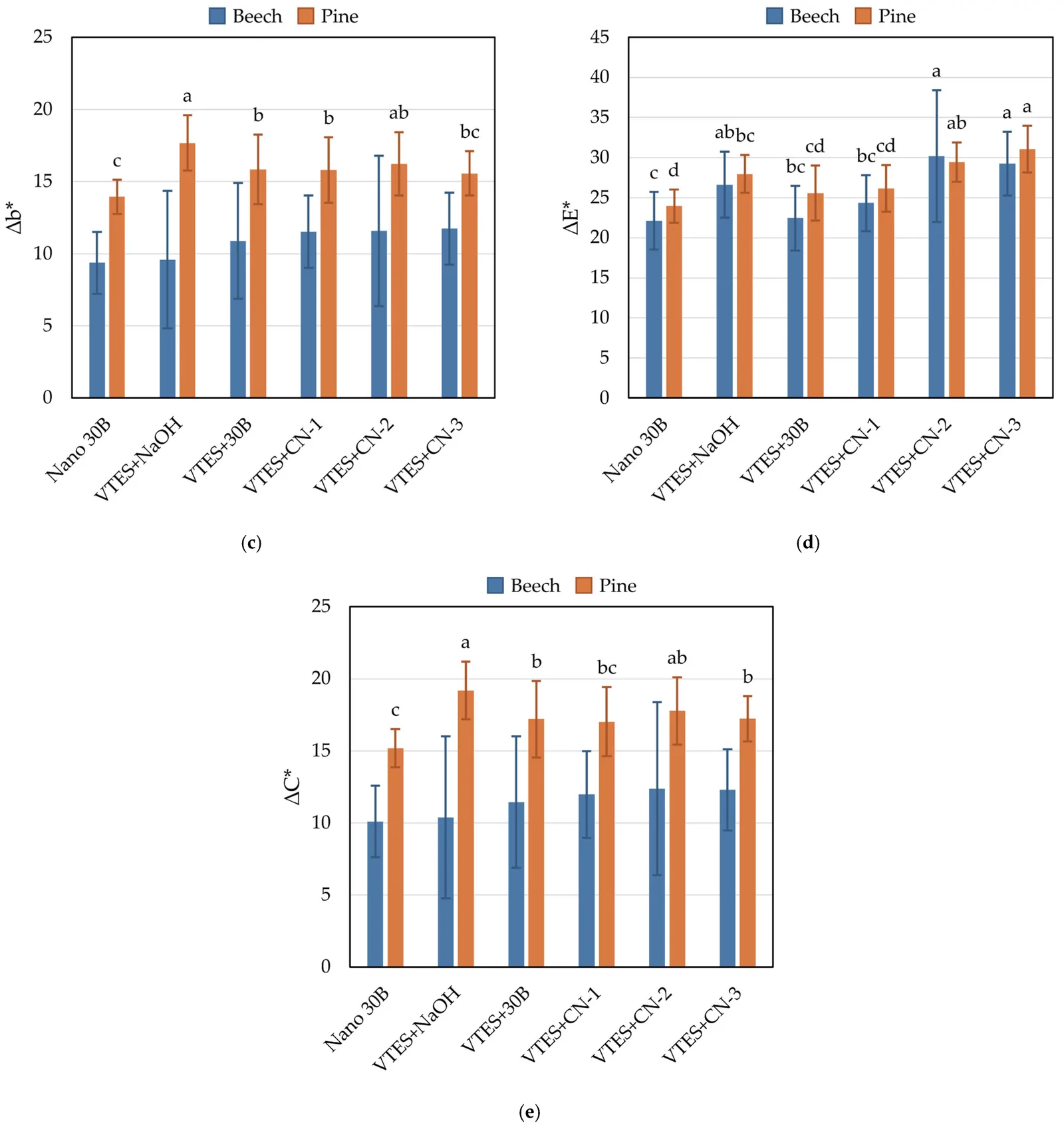
Changes in the color parameters of European beech and Scots pine following the different treatments: (**a**) lightness difference (ΔL*), (**b**) red–green coordinate difference (Δa*), (**c**) yellow–blue coordinate difference (Δb*), (**d**) total color difference (ΔE*), and (**e**) chroma difference (ΔC*). Different lowercase letters indicate significant differences among treatments within the same wood species according to Fisher’s LSD test (*p* < 0.05).

**Figure 10:**
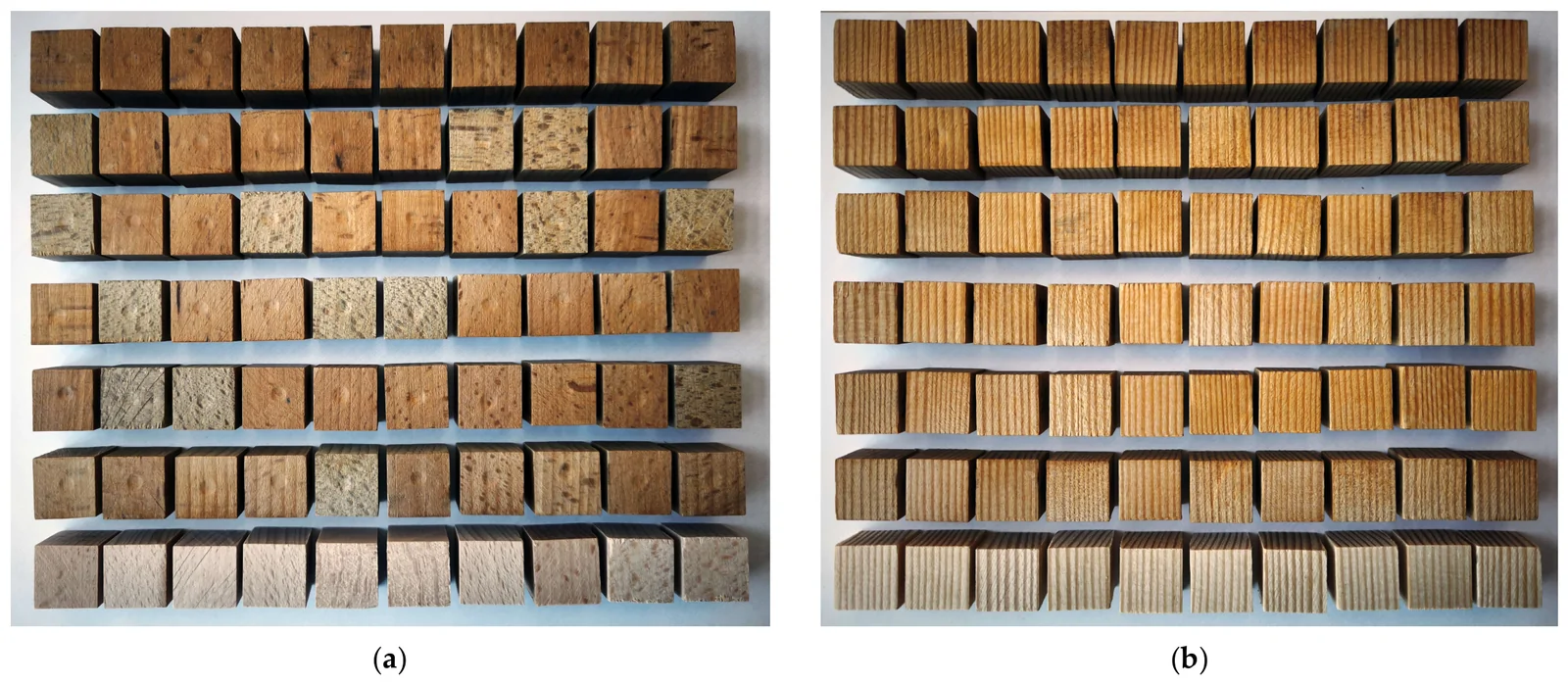
Visual appearance of (**a**) European beech and (**b**) Scots pine specimens after the different modification treatments. Rows from bottom to top represent the untreated control, Nano 30B, VTES + NaOH, VTES + 30B, VTES + CN-1, VTES + CN-2, and VTES + CN-3 groups.

The treatments caused pronounced color changes in both European beech and Scots pine (Figure 9 and Figure 10). In all treatment groups, L* decreased significantly after treatment, while a*, b*, and C* increased significantly, indicating darkening and a shift toward redder and more yellow color tones.

For European beech, ΔL* ranged from −18.99 to −27.33. The greatest darkening was observed for the VTES + CN-2 and VTES + CN-3 treatments, which also resulted in the highest ΔE* values. In contrast, no significant differences were found among the treatments for Δa*, Δb*, or ΔC*, suggesting that formulation composition mainly influenced the extent of darkening rather than the magnitude of the chromatic shift.

In Scots pine, the color response was more strongly treatment dependent. ΔL* ranged from −18.10 to −25.31, with the greatest darkening observed for VTES + CN-3, followed by VTES + CN-2. Significant treatment effects were also observed for Δa*, Δb*, and ΔC*. The highest Δa* was measured for VTES + CN-3, while VTES + NaOH resulted in the highest Δb* and ΔC* values. The greatest total color difference was observed for the VTES + CN-3 treatment.

Overall, the more pronounced color changes associated with the VTES + CN-2 and VTES + CN-3 treatments suggest that formulation composition influenced the color response. Treatment-dependent color changes have also been reported for related silica- and organosilane-based wood modification systems. Pronounced darkening was observed after in situ silica aerogel treatment of European beech and Scots pine, whereas increased particle deposition during VTES–SiO_2_–NaOH surface treatment was associated with visible whitening [12,13]. These contrasting responses suggest that the resulting color change depends more on treatment composition and the deposited material than simply on the presence of an organosilane or an inorganic phase. From utilization point of view color change is influencing the aesthetic value of wood. In case of utilization, it has to be taken into consideration if the resulting color change is acceptable or not. During outdoor applications the weathering resistance is another factor which affects the utilization. Thus, weathering performance of the investigated treatment system should be further tested.

### 3.7. FTIR Analysis

**Figure 11:**
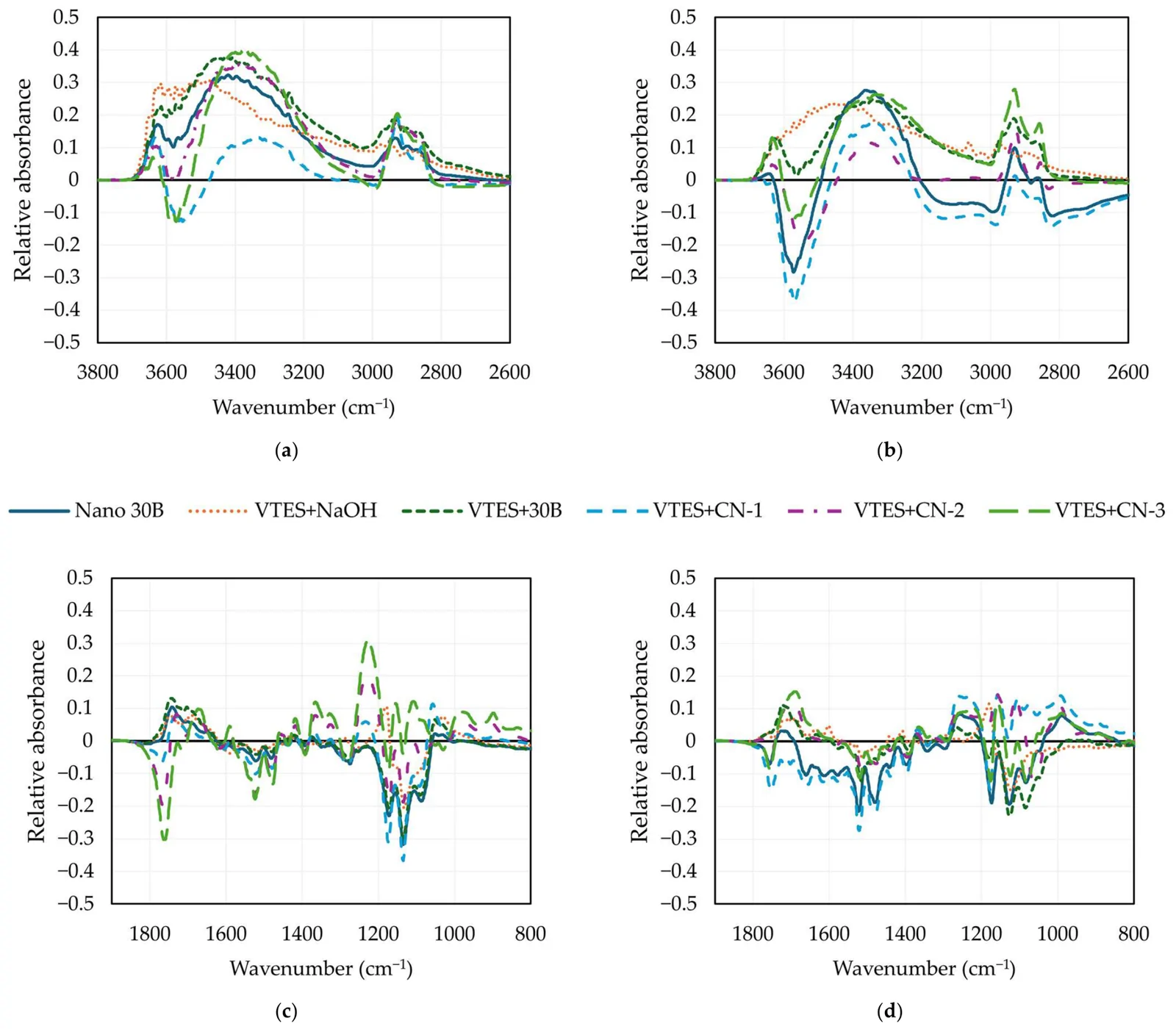
FTIR difference spectra of (**a**,**c**) European beech and (**b**,**d**) Scots pine in the 3800–2600 cm^−1^ (**a**,**b**) and 1900–800 cm^−1^ (**c**,**d**) spectral regions.

For European beech, all treatments produced broad positive differences in the 3800–2600 cm^−1^ region, with the most pronounced changes observed after the VTES + 30B, VTES + CN-2, and VTES + CN-3 treatments (Figure 11a). The VTES + CN-1 and VTES + CN-3 treatments also showed negative–positive profiles in the 3600–3300 cm^−1^ region, indicating changes in the shape and/or position of the O–H stretching band. These changes may be associated with alterations in the hydrogen-bonding environment of wood polymers, as well as with silanol groups formed during VTES hydrolysis and subsequent condensation [3,4,28]. Positive differences near 2930 cm^−1^ were observed for all treatments and were particularly pronounced in systems containing both VTES and Nano 30B. This region is associated with aliphatic C–H stretching and may therefore partly reflect organic groups originating from the treatment components [28,29,30].

In the 1900–800 cm^−1^ region, the Nano 30B, VTES + NaOH, and VTES + 30B treatments produced positive differences near 1740 cm^−1^, whereas the VTES + CN treatments showed more complex positive–negative profiles (Figure 11c). The response around 1740 cm^−1^ is consistent with changes in non-conjugated carbonyl structures mainly associated with hardwood hemicelluloses [30], although it does not necessarily indicate the formation of new carbonyl groups. Negative differences around 1520 cm^−1^ indicate changes in the relative contribution or molecular environment of lignin-related aromatic structures. The strongest treatment-dependent differences occurred between approximately 1300 and 900 cm^−1^, where vibrations of wood polysaccharides overlap with Si–O-containing structures originating from VTES-derived siloxanes and Nano 30B [28,30,31]. Consequently, the positive difference around 1050 cm^−1^ is consistent with an increased contribution from silicon-containing components, but cannot be assigned unambiguously to Si–O–Si or Si–O–C bonds.

Scots pine showed a more heterogeneous response in the 3800–2600 cm^−1^ region (Figure 11b). The VTES + NaOH and VTES + 30B treatments produced predominantly broad positive differences in the O–H stretching region, whereas Nano 30B and all three VTES + CN treatments showed negative–positive profiles between approximately 3570 and 3335 cm^−1^. These differences indicate treatment-induced changes in the distribution of hydrogen-bonded hydroxyl environments and may reflect interactions among wood hydroxyl groups, silanols, and hydroxylated nanoclay surfaces [3,4,28]. All treatments also produced positive differences near 2930 cm^−1^, while VTES + CN-3 showed the most pronounced response and an additional positive peak near 2860 cm^−1^. These changes are consistent with an increased relative contribution from aliphatic groups associated with the treatment components [28,29,30].

In the 1900–800 cm^−1^ region, most Scots pine treatments produced positive differences between approximately 1725 and 1690 cm^−1^, whereas the VTES + CN-1 treatment showed a more complex profile (Figure 11d). Compared with European beech, these carbonyl-related changes occurred at lower wavenumbers, which may be associated with differences in hemicellulose and lignin composition between hardwoods and softwoods [30]. All treatments showed negative differences near 1510–1525 cm^−1^, corresponding to the lignin-related aromatic region, although the effect was less pronounced after the VTES + NaOH treatment.

The fingerprint region provided the clearest distinction among the Scots pine treatments. Most treatments showed positive differences near 1260 cm^−1^ and negative features around 1120–1130 cm^−1^, whereas the VTES + CN-2 treatment showed distinct positive maxima at approximately 1160 and 1110 cm^−1^. Because this region contains overlapping C–O, C–O–C, and Si–O vibrations, these differences may reflect simultaneous changes in wood polysaccharides, siloxane structures, and nanoclay contributions rather than changes in a single functional group [28,30,31].

Overall, European beech showed a clearer treatment response in the carbonyl region around 1740 cm^−1^, whereas Scots pine showed stronger treatment-dependent differences in the lignin-related and fingerprint regions. These differences are consistent with the different chemical compositions of hardwoods and softwoods [30] and may also be influenced by species-dependent permeability and treatment-agent distribution [32]. Overall, the changes in the O–H, C–H, and fingerprint regions support the presence of organic and silicon-containing treatment components and indicate modifications in the molecular environment of wood polymers. However, because of the substantial overlap of the bands, the FTIR results alone do not provide conclusive evidence for the formation of covalent Si–O–C bonds between VTES and wood.

## 4. Conclusions

The combined treatment with vinyltriethoxysilane and nanoclay under alkaline conditions significantly modified the properties of European beech and Scots pine, particularly in terms of dimensional stability. Among the treatments investigated, the three-component (VTES, Cloisite 30B nanoparticles and NaOH) formulations produced the greatest reduction in volumetric swelling and the highest anti-swelling efficiency. Demonstrating that the combined system provided greater dimensional stabilization than the corresponding comparison treatments investigated in this study. Results also showed the influence of NaOH concentration on the efficiency of the treatment.

In contrast, the effects on equilibrium moisture content were less consistent and strongly depended on formulation and wood species. This indicates that the improvement in dimensional stability was not necessarily accompanied by a proportional reduction in hygroscopicity. As treatments resulting in comparable VBC values showed markedly different ASEV responses, this suggests that, in addition to volumetric bulking coefficient and equilibrium moisture content, the distribution and retention of the treatment agents may also have contributed to restricting swelling.

The combined treatment did not reduce compressive strength parallel to the grain, while the most pronounced changes in hardness were observed on the end-grain surface. All treatments caused pronounced color changes. The treatment showed promising improvements in dimensional stability and reduced fungal mass loss under laboratory conditions while maintaining compressive strength parallel to grain. Further studies are required to assess the practical applicability of the modified wood by evaluating the long-term stability of the treatment under leaching, moisture-cycling, and natural weathering conditions.

## Figures and Tables

**Table 1:** Composition of the treatment solutions.

		Nano 30B	VTES + NaOH	VTES + 30B	VTES + CN-1	VTES + CN-2	VTES + CN-3
C30B	(g)	6	-	6	6	6	6
VTES	(mL)	-	60	60	60	60	60
NaOH	(mL)	-	60 (0.1 mol/L)	-	60 (0.1 mol/L)	60 (0.4 mol/L)	60 (0.5 mol/L)
Ethanol	(mL)	600	600	600	600	600	600

## Data Availability

The original contributions presented in this study are included in the article. Further inquiries can be directed to the corresponding author.
